# Surviving the summer: foot-and-mouth disease virus survival in U.S. regional soil types at high ambient temperatures

**DOI:** 10.3389/fvets.2024.1429760

**Published:** 2024-10-24

**Authors:** Andrea L. Bessler, Serena Nayee, Rebecca Garabed, Peter Krug, John Obrycki, Luis Rodriguez

**Affiliations:** ^1^Veterinary Preventive Medicine Department, College of Veterinary Medicine, The Ohio State University, Columbus, OH, United States; ^2^Foreign Animal Disease Research Unit, Plum Island Animal Disease Center, Agricultural Research Service, United States Department of Agriculture, Greenport, NY, United States; ^3^School of Environment and Natural Resources, The Ohio State University, Columbus, OH, United States; ^4^United States Department of Agriculture, Agricultural Research Service, Manhattan, KS, United States

**Keywords:** foot and mouth disease, foreign animal disease, environment, virus survival, soil analysis

## Abstract

**Introduction:**

Foot-and-mouth disease (FMD) is one of the most economically significant global livestock diseases. In the U.S., economic optimization models run in 2011 demonstrate the highest mean epidemic impact of a potential FMD outbreak in California would occur in livestock-dense regions, resulting in national agriculture losses of $2.3 to $69.0 billion. In the case that an FMD outbreak occurred in the U.S., mass depopulation, carcass disposal, and disinfection protocols for infected premises have been designed to prevent further viral spread. Because the FMD virus (FMDV) is spread mechanically via the environment, characteristics of viral environmental stability are important. Temperature and adsorption to soil particles are reported to be the most important factors affecting general virus survival; however, how much these factors alter FMDV survival has not been tested.

**Methods:**

Soil samples were examined from typical U.S. regions containing the highest cattle population densities: Tennessee, Georgia, Nebraska, California, Pennsylvania, Kentucky, and Iowa. Soils were spiked with known quantities of FMDV and FMDV stability was evaluated over seven distinct time points between 0 hours and 12 days at incubation temperatures of 25°C and 37°C to represent a range of typical ambient temperatures during the summer. FMDV stability was quantified via virus titration.

**Results:**

Virus decayed faster at higher ambient temperatures for all soils, but decay at 25°C was faster in some soils. Consequently, areas with high ambient temperatures may have lower between-farm transmission rates, slower outbreak spread, and simpler farm decontamination.

**Discussion:**

This study provides a helpful exploration into understanding soil survival of the virus. Additional investigations into FMDV survival across different soil types will aid in developing better disinfection protocols and further refining regional viral transmission rate estimates.

## Introduction

Foot-and-mouth disease virus (FMDV; family *Picornaviridae*; genus *Aphthovirus*) is the causative agent of a severely contagious disease, foot-and-mouth disease (FMD). This virus is a single-stranded, positive-sense RNA virus consisting of seven serotypes (A, O, C, Asia 1, and South African Territories 1, 2, and 3, with serotype O being the most common worldwide), while multiple subtypes occur within each serotype (1–3). This disease is clinically characterized by fever, lameness, and debilitating vesicular lesions of the feet, tongue, snout, and teats of domestic and wild cloven-hoofed animals (2, 4, 5). FMD is one of the most economically significant global livestock diseases, as demonstrated in the 2001 FMDV outbreak that occurred in the United Kingdom. As a result of this major outbreak, losses to agriculture and the food supply amounted to about £3.1 billion nationally (6). Generally, FMD is associated with low mortality rates in adult animals. However, the global effects on agricultural industries are magnified by the combination of direct losses from decreased production and indirect losses from FMD control costs and limited access to markets (7). Simulated economic consequences of outbreaks in currently FMD-free countries and zones cause estimated monetary losses of greater than $1.5 billion a year based on one investigation (7). Specifically in the USA, epidemic simulation and economic optimization models have estimated that an FMD outbreak in California would cause national agriculture losses of $2.3–69.0 billion (8). Additionally, an epidemiological modeling study demonstrated that if an FMD outbreak were to occur in Pennsylvania, the highest mean epidemic impact would occur in Lancaster County, the county with the highest livestock density (9). These simulations indicate the greatest spread of FMDV would occur in these livestock-dense regions. Control decisions in these areas must be informed by accurate disease data and transmission rates. Early detection and intervention based on knowledge of virus survival and transmission can reduce the extent to which the disease will spread, and significantly reduce the cost of an outbreak (8). Therefore, within the United States, it is critical to develop accurate estimates of factors influencing FMDV transmission in livestock-dense regions to mitigate drastic livestock and economic losses.

Several aspects of FMDV can make it difficult to control a potential outbreak. The rapid replication cycle and structure of the virus contribute to its rather prolific nature which results in significant environmental stability, high transmission rates via contact with infected environments, and aerosol-transmitted infection (10). FMDV may be spread by aerosol, fomites, direct contact with infected animals, or ingestion by animals of contaminated feed (11–14). Specifically, fomite spread has been documented through shoes, truck tires, and other inanimate objects (14). FMDV is shed by infected animals to all excretions and secretions during the acute phase of infection and is most often found in large quantities in blister fluid, saliva, dung, and milk of disease-infected animals (15, 16). As a result, contamination of external environments or objects with any of these discharges becomes a danger to surrounding livestock and wildlife.

Since FMDV can spread mechanically, the environmental stability of the virus plays an important role in the possibility of environmentally mediated transmission. In an experiment, the contribution of environmental contamination with FMDV-infected secretions and excretions to viral transmission rates was determined via indirect contact. Uninfected calves were housed in rooms for 3 days that previously contained FMDV-inoculated calves (17). The researchers concluded that 44% of FMDV transmission was attributed to contaminated environments, therefore implying that such environments have reasonable potential to function in FMD transmission (17). Though Bravo de Rueda et al. (17) highlight an important role of the environment in transmission, their experiment was conducted in a climate-controlled setting with hard surfaces, so transmission might be expected to vary from these observations under natural conditions.

Despite the virus’ rather hardy state in nature, under experimental conditions, FMDV is most stable at near neutral or neutral pH and is extremely sensitive to acidic pH values of 6.0 and below (18). Previous studies have indicated that 90% of FMDV present was inactivated within 1 min in environmental conditions at a pH of 6.0, and at a pH of 5.0, 90% of the virus was inactivated within 1 s (19). Additionally, temperatures above 50°C typically compromise viral infectivity (20, 21), while virus maintained at around 4°C may remain active for more than 14 weeks (22–24).

While these prior studies show FMDV susceptibility to heat and pH, the extreme conditions used in these experiments are not likely to be encountered in natural farm environments. In natural settings, FMDV has been recovered from cattle stalls 14 days after removal of infected cattle, from urine after 39 days, from soil after 28 days in autumn and after 3 days in summer, and from dry hay at 22°C after 20 weeks storage (25, 26). Within cattle stalls, compared to soil outside those spaces, we expect higher organic matter content and higher pH due to fecal and urine contamination. Consequently, it is relevant to define the factors affecting FMDV viability across a range of diverse biomes to understand indirect transmission of the virus and needs for decontamination of farms after depopulation.

As an initial investigation into factors influencing the natural transmission of FMDV through the environment, we selected soil type and temperatures as key unexplored variables that should vary widely and natural settings where animals are housed. Previous studies have investigated the dependence of enterovirus survival in soil on such factors as temperature, soil moisture content, presence of aerobic microorganisms, degree of virus adsorption to the soil, silt content and clay content, soil levels of resin-extractable phosphorous, ion exchange capacity, and soil pH (27). Temperature and virus adsorption to soil have been determined to be the most important factors affecting enterovirus survival (27). Though enteroviruses are closely related to FMDV, a major difference is their resistance to acidity compared to FMDV. Therefore, more specific studies are warranted to differentiate these factors and the expected FMDV survival in soil, which have not been tested.

In this study, we examined soil samples collected from counties containing the highest cattle population densities for their United States Department of Agriculture (USDA) designated regions. Cattle compose an FMDV target group as they are noted to have the highest risk of infection as reported in previous outbreaks and demonstrated in epidemiological quantitative modeling studies (28–30). While not entirely natural, we quantified FMDV infectivity via virus titrations in soils incubated *in vitro* with FMDV. As a result, we quantified the extent to which a particular soil can preserve virus to determine how an individual environment might alter the transmission of FMDV in the event of a domestic FMDV outbreak. Incorporating this heterogeneity allows us to develop better models to predict relative FMDV spread in conditions such as those found in the United States in the summer.

## Materials and methods

### Soil collection

Soil samples were collected from seven cattle-dense states, representative of different U.S. regions defined by the USDA. Soil samples were requested from local veterinary practitioners between 1/16/15 and 3/31/15 from the most cattle-dense counties of each region, as noted in Table 1 (31). Soil samples were collected from all locations except Texas due to a lack of available resources for soil collection in that state.

**Table 1:** Cattle-dense counties and their average summer temperatures (31).

State/region	County	Average summer high temperature (°C)
Tennessee/Beltsville Area	Lincoln	31.7
Kentucky/Mid-South Area	Pulaski	29.1
Iowa/Midwest Area	Sioux	28.3
Texas/Southern Plains	Erath	33.9
Georgia/South Atlantic Area	Franklin	30.9
California/Pacific West Area	Tulare	33.3
Nebraska/Northern Plains	Cherry	29.8
Pennsylvania/North Atlantic Area	Lancaster	28.3

In total, 14 soil samples were collected from seven sites including matched pair samples from each site. Samples were collected from one representative farm within each county by local veterinarians following a written protocol. Briefly, two composite soil samples were selected one from soil inside of cattle pens and one from outside of cattle pens. All soil samples were carefully selected conditionally when it had not rained for at least one full day. Additionally, soil sampling was not permitted immediately after snow had melted, as moisture could have altered the results of later soil analyses. Areas clearly covered with manure were also avoided in the process of soil sampling, to ensure accurate analysis of the organic material contained within each soil type. Samples were shipped and maintained at ambient temperature and then split into three tubes for further testing. One sub-sample was tested for soil characteristics, the second was tested for viral survival, and the third was tested for bacterial DNA [results presented in Mills et al. (32)].

### Soil characterization

The subsample for soil characterization of each of the 14 soil samples was first dried in an oven for 12 h at 60°C, sieved to 2 mm, and split into four replicates. One replicate from each soil sample was evaluated using the following methods: Soil pH and electrical conductivity (EC), organic matter, texture, and cation exchange capacity (CEC).

*Soil pH and EC* were evaluated using a 1:10 soil-to-solution ratio using 1 g of soil and 10 g of water. Solutions were mixed by hand swirling for 30 s and allowed to settle for 10 min. Soil pH and EC were measured on the supernatant. The pH probe (pH Tester 3, Oakton Instruments) was calibrated in pH standards of 4.0, 7.0, and 10.0 (Fisher Chemical). The EC probe (EC Testr High, Oakton Instruments) was calibrated using a 1,412 μS cm^−1^ conductivity standard (Fisher Chemical). The soil-to-solution ratio can vary when measuring soil pH, so dilutions of 1:1 or 1:2 were compared, as well as 1:10.

*Estimated Organic matter* was evaluated using the loss-on-ignition technique (33). About 1 g of soil was placed in a crucible and the mass recorded. The crucible was placed in a 450°C oven for 2 h. Crucibles were removed from the oven, and they were placed in a desiccator to reach room temperature. The final mass was recorded to calculate the difference in mass before and after heating. The following equation from the Cornell Soil Health Assessment Training Manual (33) was used to convert percent mass loss (%LOI) into percent organic matter: %Organic Matter = (%LOI*0.7) − 0.23.

*Texture* was evaluated using a modified version of the texture method presented by Gugino et al. (33). The mass of soil used per sample in the texture analysis ranged from 0.33 to 4.64 g. This variation occurred due to the amount of original soil sample available for analysis. Three milliliter of a 3% sodium hexametaphosphate soap solution was added per gram of soil to a 50 mL centrifuge tube. Additional deionized water was added to the tube to reach approximately 30 mL volume. Tubes were shaken overnight. The mixture was passed through a 53 μm sieve to collect the sand fraction. Clay and silt fractions were collected in a 946 mL (32 oz) container as the solution passed through the sieve. The sand fraction was placed in crucibles. Solutions were swirled, mixed, and allowed to settle for 2.5 h. Solutions were decanted slowly until the settled silt particles began to pull away from the bottom of the cup. The silt fraction (remaining in the cup) was rinsed into crucibles. All crucibles were dried overnight at 105°C. The mass of sand and silt for each soil sample was calculated based on the mass of soil in the crucibles and the initial mass of the soil. The percent clay was calculated by taking 100% minus the sand and silt percentages. The texture was reported using the USDA texture classification system (34).

*CEC* was evaluated using a modified version of the unbuffered salt extraction method presented by Sumner and Miller (35). The extraction used a 0.2 M NH_4_Cl solution to saturate soil exchange sites with NH_4_^+^. The soil mass used for each sample ranged from 0.64 g to 2.57 g. An automated vacuum extractor was used to pull the extracting solution through the leaching tubes. One gram of shredded filter paper was used to simulate filter pulp in the leaching tube and prevent soil from moving into the final collection tube. The following extraction process was used. Ten milliliter of extracting solution was passed through the soil over 30 min. Then, 50 mL of solution was passed through the soil over 12 h. The final mass of the final collection tube was recorded. This was compared with the empty mass of each collection tube to calculate the final mass of the extracting solution. The final solution volume was calculated by dividing by the density of the solution (0.98 g mL^−1^). The solution density was calculated by measuring known volumes of the extracting solution used in this analysis. Sample solutions were transferred from the final collected tube to a 50 mL centrifuge tube. A subsample from each was poured into a 15 mL tube. This solution was analyzed for Al, Ca, K, Mg, and Na using an inductively coupled plasma-optical emission spectrophotometer (Varian ICP-OES). The results from the ICP-OES were reported in mg L^−1^. These results were converted to centimoles of charge per kg of soil (cmol_c_ kg^−1^) using the following equation adapted from Essington (36).

For a practical example, here is the calculation for Calcium:

$$\begin{array}{l} CEC\;\mathrm{in}\;cmol_{c}kg^{-1}=\left(\frac{\mathrm{cation}\;\mathrm{mg}}{L}\right)\\ {}*\left(\frac{\mathrm{final}\;\mathrm{extraction}\;\mathrm{solution}\;\mathrm{volume}\;\mathrm{in}\;L}{\mathrm{soil}\;\mathrm{mass}\;\mathrm{in}\;\mathrm{kg}}\right)*\left(\frac{1\;\mathrm{mmol}}{40.078\;\mathrm{mg}}\right)\\ {}*\frac{1\;cmol_{c}}{10\;\mathrm{mmol}} \end{array}$$

### Virus survival

#### Experimental method

The effect of 14 U.S. soil samples taken from seven locations on FMDV infectivity over seven distinct time points between 0 h and 12 days at incubation temperatures of 25°C and 37°C was determined via virus titrations. As a measure of controlling for microbial contamination, every soil sample was autoclaved to a sterile condition prior to the start of virus titration experiments. For each sample condition, 1 g of each soil type was added to a conical tube and then the soil was inoculated with 10^5^ TCID_50_ of FMDV serotype A (A_24_ Cruzeiro) in 1 mL of infection media (DMEM, 1X Hepes buffer solution, 1X Antibiotic-Antimycotic solution) then the tube was sealed, mixed by inversion and incubated at the indicated temperature for the indicated time in cell culture incubators. Control samples contained either only 1 mL of inoculum alone or only 1 g of soil with 1 mL of infection media and no virus. Soil interference with virus recovery was determined by freezing inoculated soil samples immediately (zero-hour timepoint) and comparing them to virus controls with no soil. For each condition, two replicates of soil and control samples were incubated at either 25°C or 37°C and were collected at 2 h, 1 day, 2 days, 5 days, 7 days, 9 days, and 12 days. At the indicated time after inoculation, the samples were moved to a −70°C freezer for storage until processing.

Virus titrations of all soil samples and control samples were performed on the same day to prevent assay-to-assay variation. Soil and control samples were removed from the freezer, thawed and 10-fold serially diluted six times in infection media. Each dilution for each condition was added to four replicate wells of 96-well plates containing confluent LFBK-αVβ6 cells (38). These cultures were incubated at 37°C in a CO_2_ incubator for 72 h. At 72 h post-infection, titration plates were stained with 100 μL/well of tissue fixative (HistoChoice®, Amresco, Inc) containing crystal violet to visualize the cytopathic effect. For each soil type or control at every given time point, a TCID_50_ value was calculated via the Spearmann-Karber method to quantify the infectivity of the virus (39).

#### Data analysis

Version 4.2.2 R statistical software was used for all statistical analyses. Initially, log TCID_50_ values obtained from virus titrations were plotted to visualize virus survival as a function of time for the different soil types (by state collected) by location (inside the pens vs. outside the pens) and incubation temperatures to provide an overview of viral survival among the samples collected. An association between virus titration and location of sample collection (inside vs. outside) using the Kruskal-Wallis rank sum test was investigated, as well as explored for model selection. Subsequently, the data for the two incubation temperatures were kept distinct from each other and analyzed separately. A covariate correlation matrix was examined to determine the effect of various soil characteristics (temperature, pH, electrical conductivity [EC], estimated organic matter [EOM], and soil texture [% of sand, silt, clay]) on virus titrations. Furthermore, soil characteristics inside versus outside of pens were compared with a T-test using the base ‘stats’ package in R statistical software (R Foundation for Statistical Computing, Vienna, Austria). Upon exploratory investigation, the data failed tests to demonstrate linearity, and other models to portray the shape of the data were explored, including spline and logarithmic relationships. Linear regression, spline regression, and logarithmic models were examined to determine the model of best fit for each incubation temperature using the ‘lm’ (*Stats package* Version 4.2.2; (37), ‘lm ~ bs’ (*Splines package Version 4.2.2;* (40), and ‘log’ (*Base package Version 4.2.2;* (41) functions in R statistical software (R Foundation for Statistical Computing, Vienna, Austria) to estimate the effects of virus survival over time, based on incubation temperature (output provided in Appendix). The model shape that best fit the data was a spline curve and this was used to continue the analysis. The spline model is a more appropriate method as it breaks the data into segments and those piecewise curves allow for greater flexibility of the data, as well as departures from linearity. To assess the effects of soil characteristics on viral survival at each incubation temperature, a forward stepwise selection method comparing adjusted R-squares, Akaike Information Criteria (AIC), and Bayesian Information Criteria (BIC) was used. Two base models were used for analysis that included the log TCID_50_ value over time for each incubation temperature. For each base model, each additional soil characteristic was added sequentially as a model covariate. The order of inclusion of covariates for the forward stepwise selection for the model at 25°C was as follows: pH, EC, EOM, sand, silt, clay, clay + EC, clay + EC + pH, clay + EC + EOM, EC + EOM + sand, EC + EOM. Whereas the order of inclusion of covariates for the 37°C base model was as follows: pH, EC, EOM, sand, silt, clay, silt + sand, silt + EC, silt + EOM. Summary tables for each model selection are provided in Supplementary Tables S1.2, S1.3, respectively. The best-fitting model was selected based on comparing the adjusted R-squared closest to 1, lowest AIC, and lowest BIC, and the resulting predicted survival lines were plotted in R [*Tidyverse* package Version 4.2.2; (45) & *Stats package* Version 4.2.2; (37)].

## Results

Soils were characterized according to pH, electrical conductivity, estimated organic matter, texture, texture class, calcium, potassium, magnesium, and sodium content, and soil cation exchange capacity. Iowa and California samples had the highest clay content. Because the degree of virus adsorption is proportional to silt and clay content, Iowa and California samples were expected to show a higher degree of virus adsorption over time. Iowa samples did retain a higher amount of virus for a longer period in comparison to most other samples, based on TCID_50_ values.

### Soil characterization

All soil types were divided according to location (state of collection and inside versus outside of cattle pens). The characteristics of each soil type are shown below in Table 2. As expected, samples collected from inside pens had a higher pH than those collected outside of pens (mean difference = 1.8, T-test *p*-value = 0.0019, Version 4.2.2 R statistical software). Other characteristics were also greater inside pens, but with decreasing significance—mean differences: 5.86, 0.74, 4.57, and 7.43; and *p*-values: 0.0813, 0.0977, 0.2075, and 0.2689—for organic matter, EC, clay, and CEC, respectively. As the soil analyses were done with limited volumes of soil and have some analytical variability, the true significance of differences is likely somewhat less than what is reported here.

**Table 2:** Characteristics of soil samples.

State	Location	pH	Electrical conductivity (mS/cm)	Estimated Organic Matter (%)	Texture – Sand (%)	Texture – Silt (%)	Texture – Clay (%)	Texture Class	Ca cmolc/kg	K cmolc/kg	Mg cmolc/kg	Na cmolc/kg	CEC Sum cmolc/kg
California	Outside	7.1	0.5	6	42	24	34	Clay loam	7	7	12	12	38
California	Inside	8.9	4.1	31	57	6	37	Sandy Clay	20	21	33	35	109
Nebraska	Outside	6.2	0.1	3	95	1	4	Fine Sand	1	1	2	2	7
Nebraska	Inside	7.8	0.1	4	64	2	34	Sandy clay loam	3	3	4	5	14
Iowa	Outside	6.9	0.0	2	3	56	41	Silty Clay	8	9	14	15	45
Iowa	Inside	8.6	1.3	15	43	33	24	Loam	8	8	12	13	41
Georgia	Outside	4.0	0.1	5	69	20	12	Sandy loam	3	3	5	5	15
Georgia	Inside	7.9	0.3	8	76	11	13	Sandy loam	4	4	7	7	22
Tennessee	Outside	6.6	0.3	8	49	38	13	Loam	8	8	14	14	45
Tennessee	Inside	7.3	0.2	6	38	44	18	Loam	5	5	8	8	25
Pennsylvania	Outside	6.3	0.3	11	47	30	23	Loam	9	10	16	17	51
Pennsylvania	Inside	8.3	0.6	12	55	21	25	Sandy clay loam	7	7	11	12	36
Kentucky	Outside	5.9	0.1	4	51	30	19	Loam	3	3	5	5	16
Kentucky	Inside	6.8	0.0	4	43	43	27	Clay loam	4	4	7	7	22

### Virus survival

**Figure 1:**
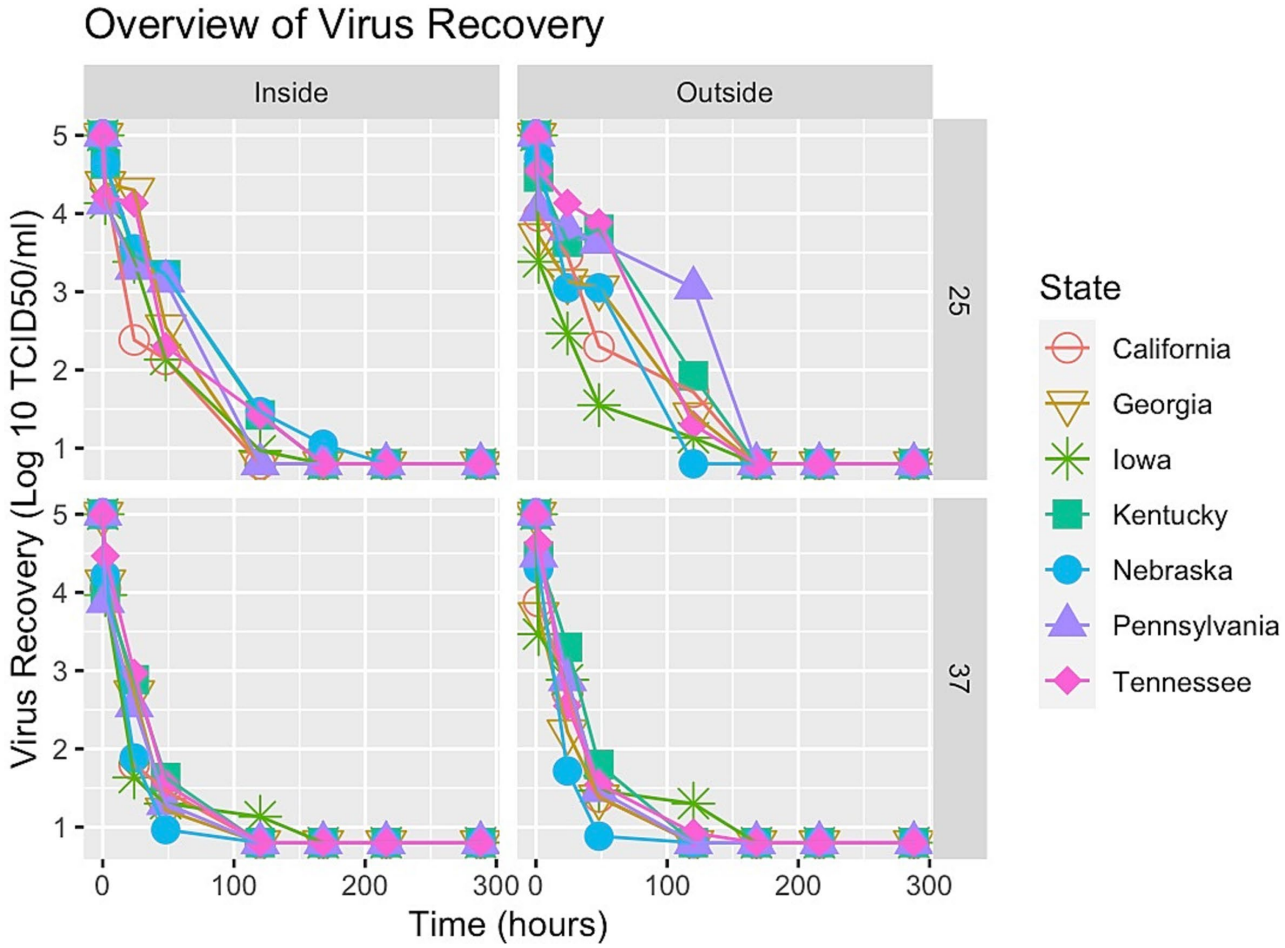
Overview of all soils.

Virus survival for each sample was plotted at both incubation temperatures (25°C and 37°C) for each location (inside the pen and outside the pen) shown in Figure 1. Viral inactivation did not appear linear over time, rather some tailing (biphasic curves) was observed. Therefore, the spline shape was examined to partition the segments of time and describe the nonlinear distributions of virus inactivation over those segments of time. To determine which soil parameters had the largest effect on virus survival, covariates were added to the spline model using the stepwise forward selection method.

The covariates indicating the strongest correlation with virus titration quantities were EC and EOM [output table provided in Appendix]. An association between virus titration and location of sample collection (inside vs. outside) using the Kruskal-Wallis rank sum test was investigated but did not produce significant evidence of association (*p*-value >0.05).

**Figure 2:**
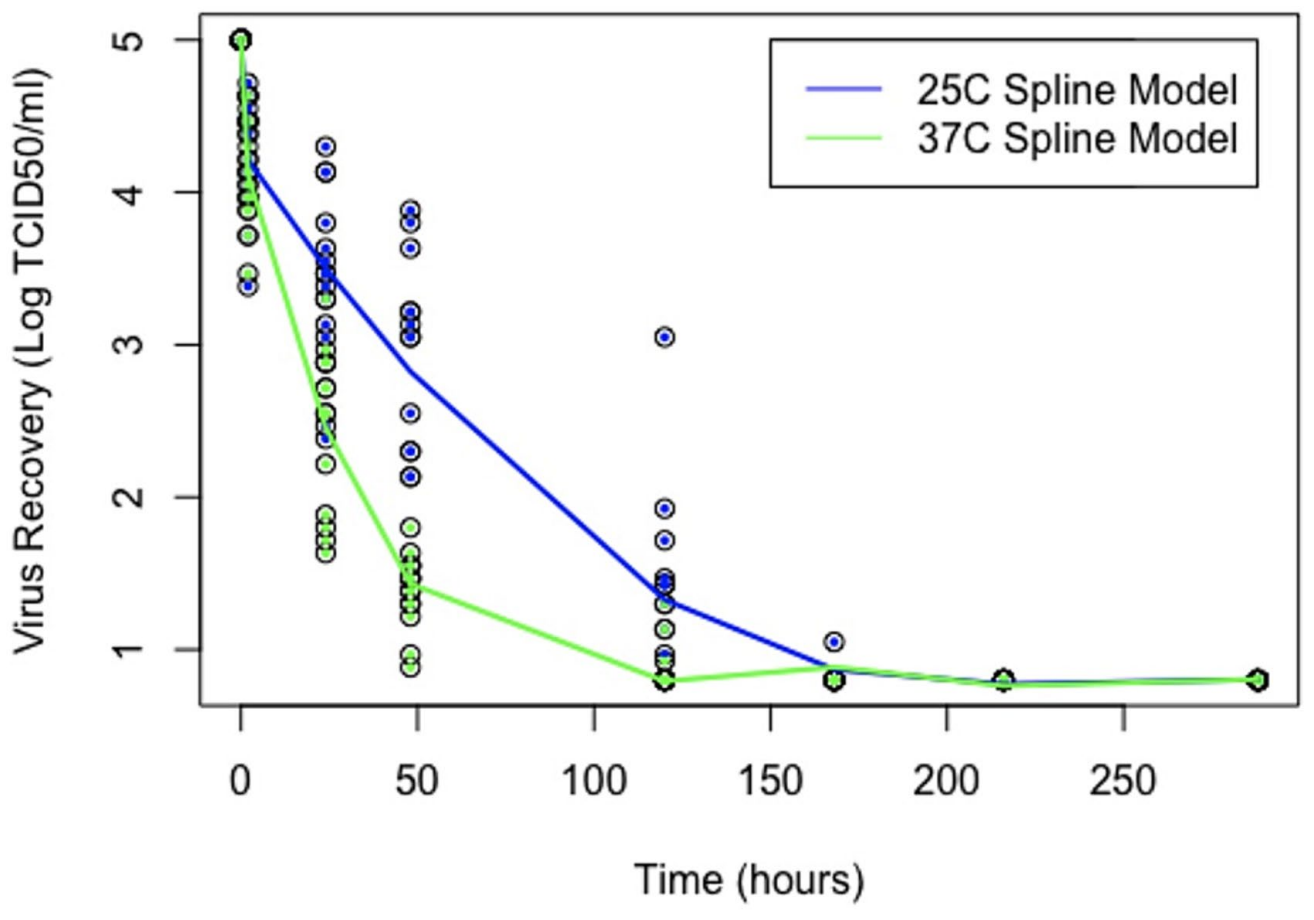
Virus survival in soil over time. Points represent observed data and lines represent model estimates, using the “spline” and package Version 4.2.2.

**Figure 3:**
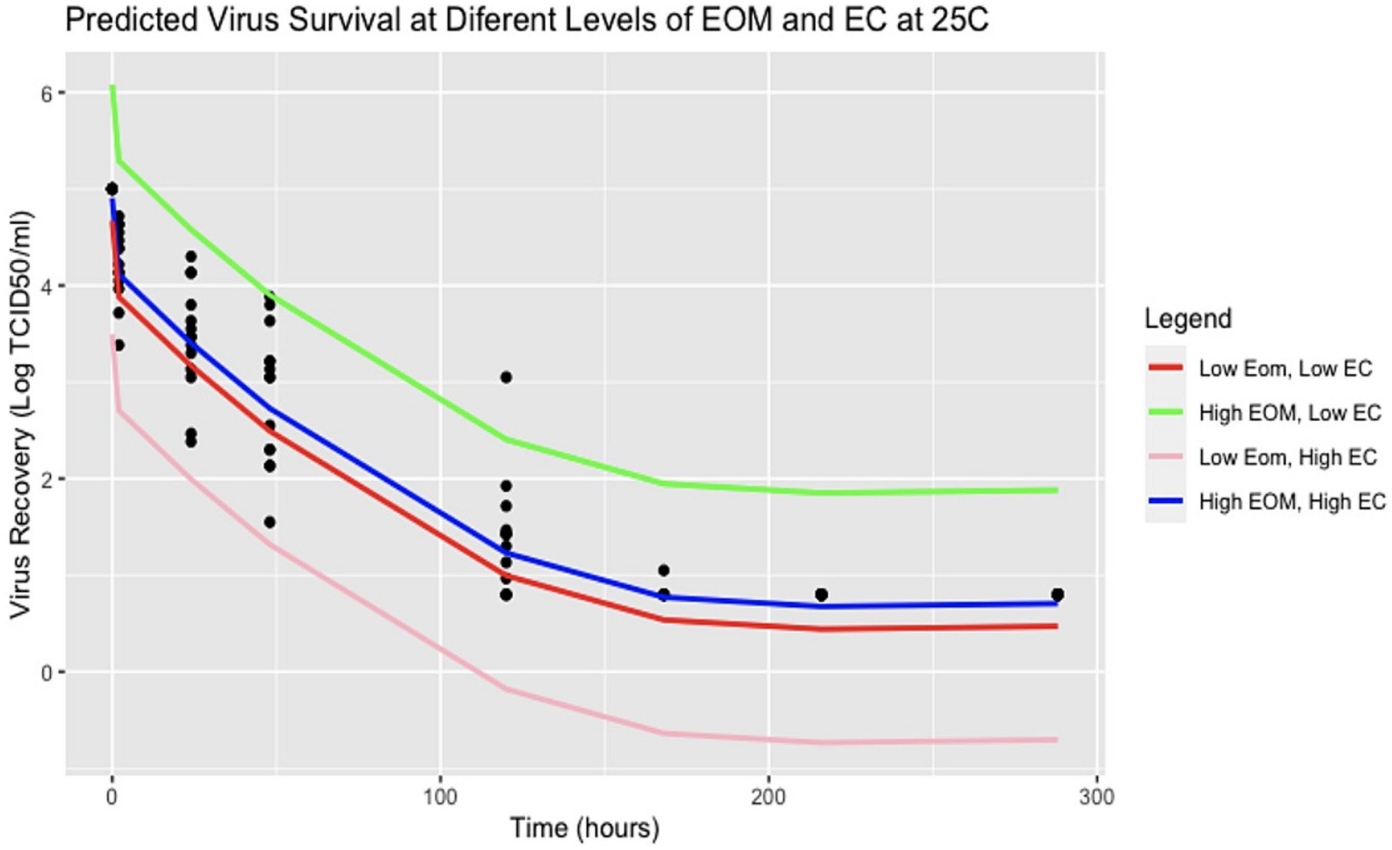
Predicted virus survival with various parameters of EC and EOM at 25°C. Points represent observed data and lines represent model estimates using R Version 4.2.2.

**Figure 4:**
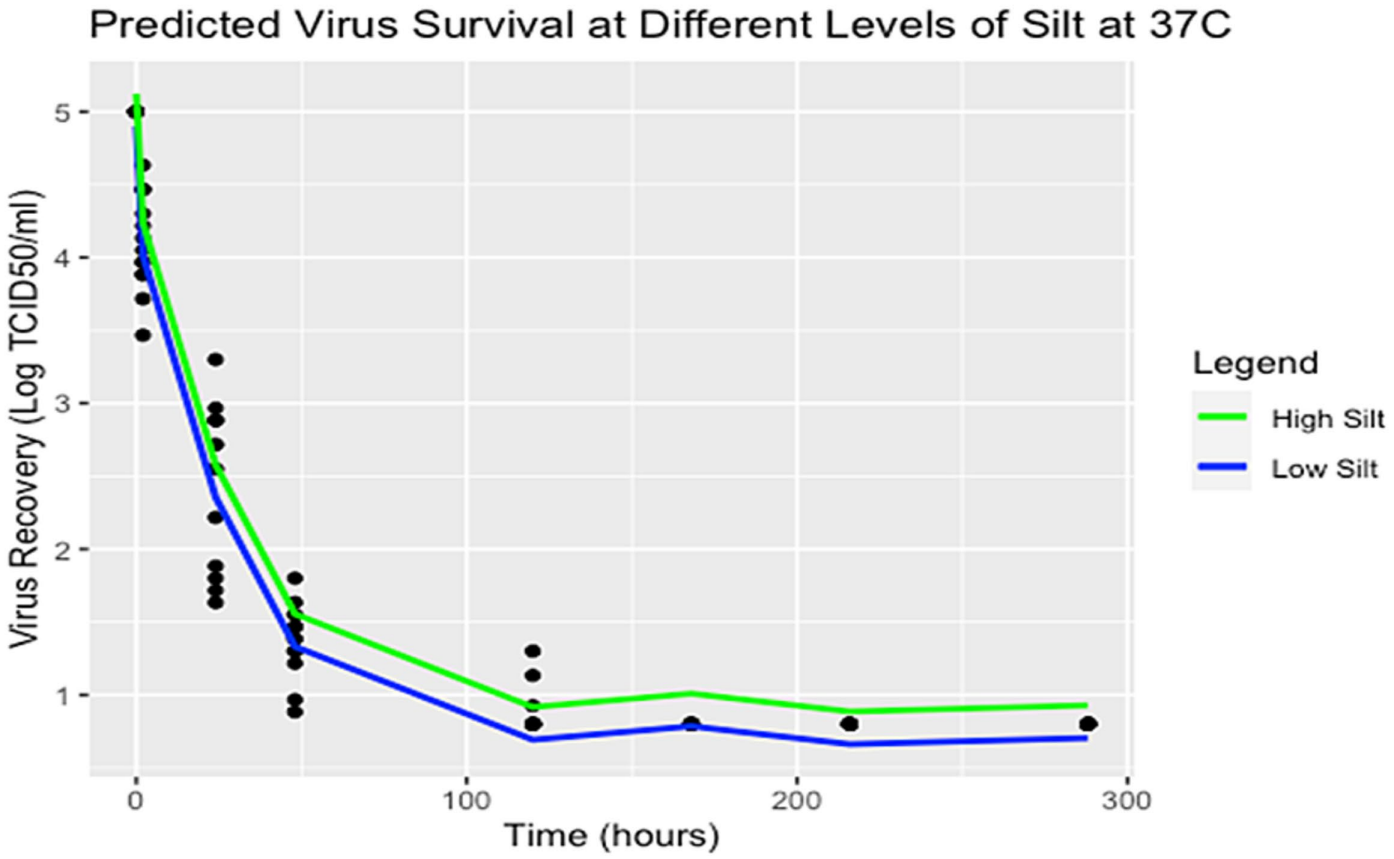
Predicted virus survival with various parameters of silt at 37°C. Points represent observed data and lines represent model estimates using R Version 4.2.2.

The forward stepwise selection summaries of the spline regression models for each temperature are reported in Appendix. For 25°C, the most significant soil characteristics affecting virus survival were EC and clay. However, the model including the covariates EC and EOM was selected based on a combination of the adjusted R-squared closest to 1 (0.9432), lowest AIC value at 117.4989, and lowest BIC value at 141.9654. For 37°C, the most significant soil characteristics affecting virus survival were silt and clay. However, the model including silt was selected as the best fit for the data based on a combination of the Adjusted R-squared closest to 1 (0.9776), the lowest AIC value of 5.618074, and the lowest BIC value at 27.36607. Details for each of the best-fitting models are shown in Tables 3, 4. A spline regression curve was fit to the data based on each model, shown in Figure 2. Adjusting the parameters of the model covariates to predict virus survival over time for each temperature is shown in Figures 3, 4.

**Table 3:** Coefficients of the best-fit spline regression model for 25°C including time, EOM, and EC to describe the decay of FMDV expressed as logTCID50.

Covariate	Estimate	Standard error	*p*-value
Intercept	4.85872	0.13638	<2e−16
Spline of time (h)	−4.19819	0.14794	<2e−16
EOM	0.04052	0.01820	0.02813
EC	−0.35247	0.12703	0.00655

**Table 4:** Coefficients of the best-fit spline regression model for 37°C including time, EOM, and EC to describe the decay of FMDV expressed as logTCID50.

Covariate	Estimate	Standard error	*p*-value
Intercept	4.895606	0.073174	<2e−16
Spline of time (h)	−4.195579	0.090152	<2e−16
Silt	0.004072	0.001401	0.00445

Overall, both models show a negative relationship between virus and time where virus titers decreased over time. For the spline model at 25°C, it appears that EOM has a protective quality for virus survival over time, whereas EC is destructive for virus over time. The two models are plotted using average values of EC and EOM for 25°C and the percentage of silt for 37°C is plotted in Figure 2. Spline prediction curves for different levels of EC (high at 4.1 and low at 0.1) and EOM (high at 31 and low at 2) are demonstrated in Figure 3. For the spline model at 37°C, silt has a minimal protective quality. Spline prediction curves for high (56%) and low silt (1%) are plotted in Figure 4.

As seen in Figure 3, at 25°C high EOM appears to be protective while higher EC decreases virus survival. Possible locations where these parameters might be observed, and impact virus survival can be found in the samples collected. The sample with a high EOM of 31 was California, while the sample with a low EOM of 2 was Iowa. The state with an observed high EC of 4.1 was California, whereas the state with a low EC of 0.1 was Nebraska. Additionally, as demonstrated in the predicted survival plot for 37°C (Figure 4), high proportions of silt have a minor protective effect on viral survivability. The state with a sample containing a high silt percentage in the soil was Iowa, whereas the sample with a low silt percentage was Nebraska. Nearly all virus infectivity was absent at 168 h (7 days), but the model predicts that areas with high EOM and low EC might have extended survival at 25°C.

## Discussion

The environmental spread of FMDV is often poorly described in the context of soil retention of the virus. Previous studies have investigated the general role of soil in virus survival and the role of differing soil types in virus survival. However, the characteristic impact of differing soil types from cattle-dense regions on FMDV survival, specifically, has yet to be determined. Therefore, this study established a trend in virus survival as a function of time, temperature, and soil type, to guide further studies that could aid in the development of disease control and prevention protocols against potential FMDV outbreaks in the United States.

Thermal inactivation efficiency of FMDV *in vitro* is a function of time and temperature with viral inactivation occurring linearly on the log scale over time (21). However, Kamolsiripichaiporn et al. (21) observed some biphasic curves and tailing of FMDV survival even at high temperatures. Like Kamolsiripichaiporn et al., our study also demonstrated a nonlinear relationship of virus inactivation, so alternative shapes to linear were analyzed to describe the data. A spline curve was utilized in this study as the spline method for compartmentalization of the data demonstrates greater flexibility and incorporates nonlinear relationships.

In this study, analysis of virus titers suggested that the difference between incubation temperatures of 25°C and 37°C had more of an impact on retention of infectious virus in soil than did soil characteristics themselves. As many previous studies have been done either at higher (24) or lower (17, 25) temperatures, this study fills a gap in the literature relating to relative virus survival at summer temperatures in the United States. Furthermore, in a review conducted by Mielke and Garabed (42), some of the same parameters and their effects on FMDV survival were examined. This review showed that there was increased survival at lower temperatures (−6°C) and lower relative humidity. Additionally, higher RH and higher temperature (37°C) also increased survival. While 37°C is higher than the average daily high temperatures in the US in summer, it is not unusual for temperatures to reach this level in cattle-dense regions, so it provides a useful worst (or best) case scenario of FMDV survival, worst for FMDV and best for disease control.

As the soil analyses were done with limited volumes of soil and have some analytical variability, the true significance of differences is likely somewhat less than what is reported here. While the methodology for model selection is acknowledged to be biased, these results can help guide future studies, especially with additional samples from different areas within the cattle-dense states themselves as the authors acknowledge the limitation of sampling only one area within the selected state. However, our analyses generally suggest virus survival persisted longer over time at 25°C incubation temperatures, especially for soil collected outside of cattle pens (Figure 1). Further, at 25°C, EOM is the soil characteristic that contributes to more virus protection, whereas EC had a destructive effect on virus survival (Table 3; Figure 3). On the other hand, while the samples with an incubation temperature of 37°C experienced quicker virus inactivation, those with a higher percentage of silt content showed a protective quality for virus survival (Table 4; Figure 4). Interestingly, soils with higher levels of silt (and clay) tend to have higher levels of organic matter (43), so two of the significant covariates found in our analysis might be even more important for virus survival and could be investigated further.

High silt content has been shown to increase virus adsorption for related viruses (27). Though our findings support the idea that soil types with higher silt content may also retain live virus for a longer period, soil samples from Iowa, Tennessee, and Kentucky with high silt content did not show similar FMDV survival. This may relate to the small amounts of soil used in the soil characterization or indicate another aspect of the samples not measured. Silt-rich areas and areas with soils that have higher levels of organic matter do exist commonly within the Southeastern and Eastern regions of the United States, Kentucky, and Tennessee, particularly among them (44). A deeper examination of silt and perhaps clay content can be a topic of further investigation as well as other environmental conditions such as relative humidity.

Sometimes percent clay content correlates with EOM, which was found to be significant at 25°C in our analysis, but in the areas inside enclosures that contain highly modified soil with amendments, the high level of organic matter contributed by cattle may mean that our study observed an EOM effect not a hidden clay effect, so EOM itself may need further exploration. Additionally, the very high measure of EOM (31%) found inside the cattle enclosure in California suggests that the characteristics of the matrix under cattle may not be those of natural soil, and the mixture of soil, manure and some type of compost is more characteristic of the environment inside animal enclosures, as opposed to the natural soil matrix. This presentation of high EOM and soil composition may require additional methods for characterization to understand how the matrix may protect or destroy FMDV.

Overall, the relevance of these findings remains significant in the context of developing estimates to account for heterogeneities in the transmission of potential FMDV outbreaks within the United States. For example, areas in which seasonal temperatures are at or below 25°C should be monitored more closely for a longer period following depopulation activities and might be expected to have higher disease transmission rates than hotter areas. Areas with high ambient temperatures may rely more on direct animal-to-animal transmission rather than environmentally mediated transmission. However, adsorption to organic matter or silt/clay particles in some soils could contribute to additional environmentally mediated transmission. In the case of less environmentally mediated transmission, transmission rates may be slower than predicted and more sporadic over space due to animal movements, which could reduce the effectiveness of control measures based on the radius from infected premises. More work on the mechanisms behind FMDV survival in soils may aid in determining better disinfection protocols and better characterization of variability in transmission. It is also possible to expand the application of these results to areas outside of the United States with similar soil and temperature characteristics. Temperatures of 25°C and 37°C are quite common in areas with endemic FMDV circulation, so the finding of variable survival in this temperature range is relevant to endemic disease control efforts. However, further studies that expand the application of these results should test the effects of differing soil types on viral infectivity with a greater number and volume of soil subsamples to characterize each soil type and a greater variety of FMDV strains as individual strains may vary in their environmental survival.

## Data Availability

The original contributions presented in the study are included in the article/Supplementary material, further inquiries can be directed to the corresponding author.
