# Supplementary material for: Surviving the summer: foot-and-mouth disease virus survival in U.S. regional soil types at high ambient temperatures
Source: Front Vet Sci. 2024 Oct 24;11:1429760. doi: 10.3389/fvets.2024.1429760 (PMC11541954; doi:10.3389/fvets.2024.1429760)
Supplement: Supplementary file 1 [file Data_Sheet_1.DOCX]

Supplementary Material

# Model Selection

**Table S1.1: Model selection table to determine shape that best fits the data**

| **Model (VT~Time)** | **P-Value** | **Adjusted R Squared** | **AIC** | **BIC** |
| --- | --- | --- | --- | --- |
| Linear regression at 25C | < 2.2e-16 | 0.7699 | 268.3957 | 276.5512 |
| Spline regression at 25C | < 2.2e-16 | 0.9388 | 123.8532 | 142.8827 |
| Log at 25C | < 2.2e-16 | 0.7447 | 280.0241 | 288.1796 |
| Linear regression at 37C | < 2.2e-16 | 0.5861 | 327.3267 | 335.4822 |
| Spline regression at 37C | < 2.2e-16 | 0.976 | 12.28746 | 31.31695 |
| Log at 37C | < 2.2e-16 | 0.8838 | 185.0407 | 193.1962 |

**Table S1.2: Spline regression model selection summary for 25°C**

| **Spline Regression Model for 25°C** | **p-value for covariate** | **P-Value for Model** | **Adjusted R Squared** | **AIC** | **BIC** |
| --- | --- | --- | --- | --- | --- |
| pH | 0.108 | < 2.2e-16 | 0.9398 | 123.0808 | 144.8288 |
| EC | 0.0286 | < 2.2e-16 | 0.941 | 120.7149 | 142.4629 |
| EOM | 0.138 | < 2.2e-16 | 0.9395 | 123.4979 | 145.2458 |
| Sand | 0.177 | < 2.2e-16 | 0.9393 | 123.8955 | 145.6435 |
| Silt | 0.733 | < 2.2e-16 | 0.9383 | 125.729 | 147.477 |
| Clay | 0.0244 | < 2.2e-16 | 0.9412 | 120.4231 | 142.1711 |
| Clay + EC | 0.115 0.137 | < 2.2e-16 | 0.9419 | 120.0337 | 144.5002 |
| Clay + EC + pH | 0.134 0.180 0.934 | < 2.2e-16 | 0.9413 | 122.0263 | 149.2113 |
| Clay + EC + EOM | 0.2788 0.0270 0.0616 | < 2.2e-16 | 0.9433 | 118.2174 | 145.4024 |
| *EC + EOM +* Sand | *0.00769 0.03506 0.15988* | *< 2.2e-16* | *0.9437* | *117.3405* | *144.5255* |
| **EC** + **EOM** | 0.00655 0.02813 | < 2.2e-16 | 0.9432 | 117.4989 | 141.9654 |

**Table S1.3: Spline regression model selection summary for 37°C**

| **Spline Regression Model for 37°C** | **p-value for covariate** | **P-Value for Model** | **Adjusted R Squared** | **AIC** | **BIC** |
| --- | --- | --- | --- | --- | --- |
| pH | 0.33 | < 2.2e-16 | 0.976 | 13.26888 | 35.01687 |
| EC | 0.167 | < 2.2e-16 | 0.9762 | 12.24407 | 33.99206 |
| EOM | 0.271 | < 2.2e-16 | 0.976 | 12.99086 | 34.73885 |
| Sand | 0.0383 | < 2.2e-16 | 0.9767 | 9.686003 | 31.43399 |
| **Silt** | 0.00445 | < 2.2e-16 | 0.9776 | 5.618074 | 27.36607 |
| Clay | 0.832 | < 2.2e-16 | 0.9758 | 14.23916 | 35.98715 |
| Silt + Sand | 0.0349 0.4053 | < 2.2e-16 | 0.9775 | 6.868637 | 31.33513 |
| Silt + EC | 0.0115 0.6062 | < 2.2e-16 | 0.9774 | 7.330504 | 31.79699 |
| Silt + EOM | 0.00859 0.76000 | < 2.2e-16 | 0.9774 | 7.517091 | 31.98358 |

# Covariate Correlation


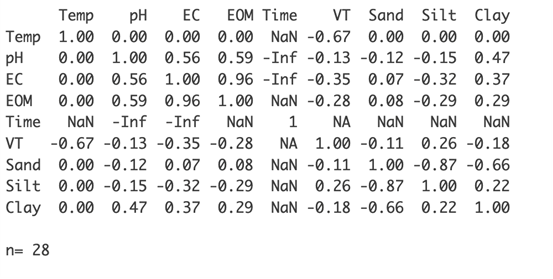


**Figure S2.1:  Covariate correlation matrix**


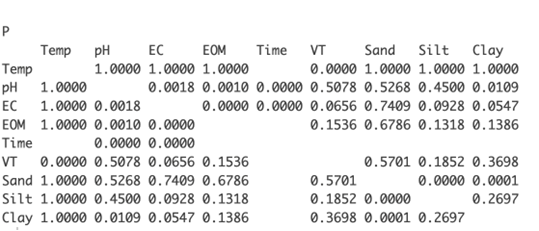


**Figure S2.2: Associated p-values for covariate correlation matrix**

# FMDV Survival by State

**
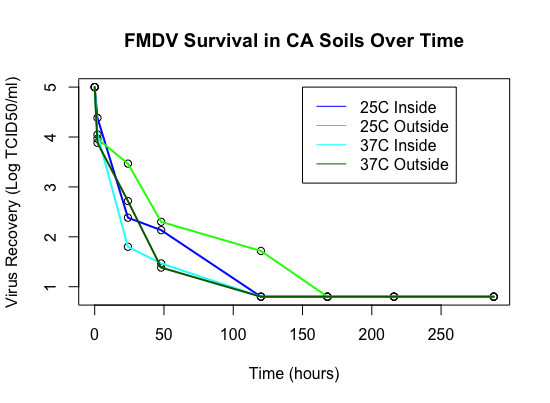
**

**Figure S3.1: FMDV survival in CA soils over time**

**
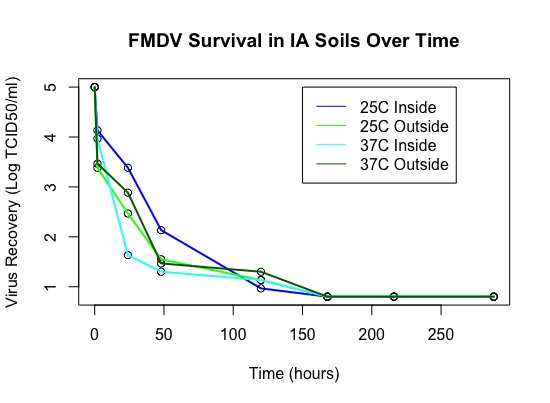
**

**Figure S3.2: FMDV survival in IA soils over time**

**
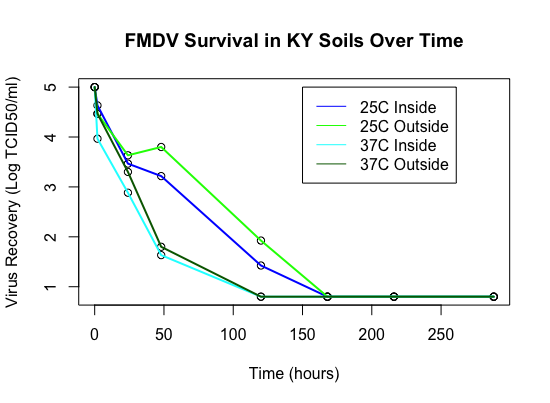
**

**Figure S3.3: FMDV survival in KY soils over time**

**
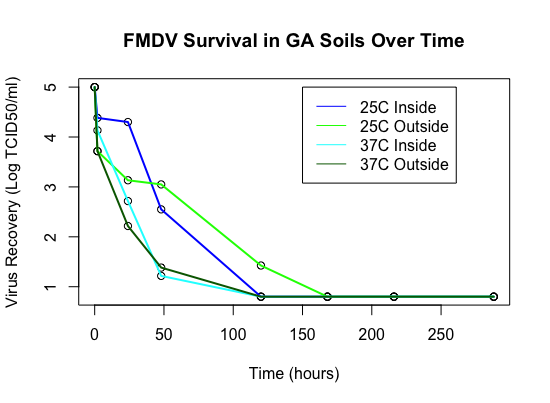
**

**Figure S3.4: FMDV survival in GA soils over time**

**
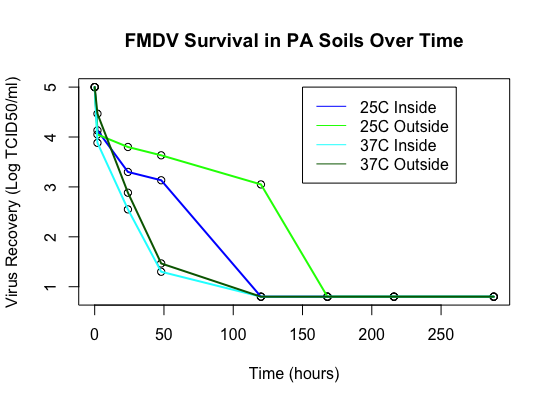
**

**Figure S3.5: FMDV survival in PA soils over time**

**
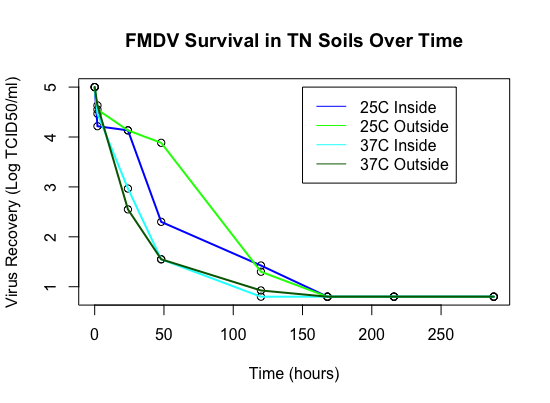
**

**Figure S3.6: FMDV survival in TN soils over time**

**
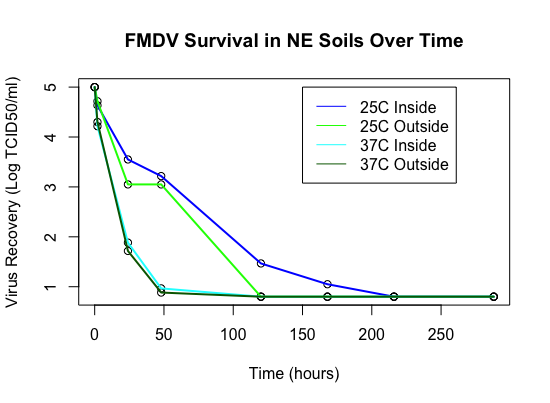
**

**Figure S3.7: FMDV survival in NE soils over time**
